# Dosimetric validation of the couch and coil model for high-field MR-linac treatment planning

**DOI:** 10.1016/j.zemedi.2023.02.002

**Published:** 2023-03-27

**Authors:** Hans Lynggaard Riis, Rasmus Lübeck Christiansen, Nina Tilly, David Tilly

**Affiliations:** a Odense University Hospital, Department of Oncology, Odense, Denmark; b University of Southern Denmark, Department of Clinical Research, Odense, Denmark; c Medical Radiation Physics, Department of Immunology, Genetics and Pathology, Uppsala University, Uppsala, Sweden; d Elekta Instrument AB, Stockholm, Sweden; e Medical Physics, Akademiska Sjukhuset, Uppsala, Sweden

**Keywords:** MRIgRT, couch, coil, attenuation, cryostat, magnetic field, TPS, dosimetry, ion chambers

## Abstract

**Purpose:**

The precision of the dose delivery in radiation therapy with high-field MR-linacs is challenging due to the substantial variation in the beam attenuation of the patient positioning system (PPS) (the couch and coils) as a function of the gantry angle. This work aimed to compare the attenuation of two PPSs located at two different MR-linac sites through measurements and calculations in the treatment planning system (TPS).

**Methods:**

Attenuation measurements were performed at every 1° gantry angle at the two sites with a cylindrical water phantom with a Farmer chamber inserted along the rotational axis of the phantom. The phantom was positioned with the chamber reference point (CRP) at the MR-linac isocentre. A compensation strategy was applied to minimise sinusoidal measurement errors due to, e.g. air cavity or setup. A series of tests were performed to assess the sensitivity to measurement uncertainties. The dose to a model of the cylindrical water phantom with the PPS added was calculated in the TPS (Monaco v5.4 as well as in a development version Dev of an upcoming release), for the same gantry angles as for the measurements. The TPS PPS model dependency of the dose calculation voxelisation resolution was also investigated.

**Results:**

A comparison of the measured attenuation of the two PPSs yielded differences of less than 0.5% for most gantry angles. The maximum deviation between the attenuation measurements for the two different PPSs exceeded ±1% at two specific gantry angles 115° and 245°, where the beam traverses the most complex PPS structures. The attenuation increases from 0% to 25% in 15° intervals around these angles. The measured and calculated attenuation, as calculated in v5.4, was generally within 1-2% with a systematic overestimation of the attenuation for gantry angles around 180°, as well as a maximum error of 4-5% for a few discrete angles in 10° gantry angle intervals around the complex PPS structures. The PPS modelling was improved compared to v5.4 in Dev, especially around 180°, and the results of those calculations were within ±1%, but with a similar 4% maximum deviation for the most complex PPS structures.

**Conclusions:**

Generally, the two tested PPS structures exhibit very similar attenuation as a function of the gantry angle, including the angles with a steep change in attenuation. Both TPS versions, v5.4 and Dev delivered clinically acceptable accuracy of the calculated dose, as the differences in the measurements were overall better than ±2%. Additionally, Dev improved the accuracy of the dose calculation to ±1% for gantry angles around 180°.

## 1 Introduction

Over the last years, linear accelerators (linacs) with integrated magnetic resonance imaging (MRI) (MR-linacs) have become clinically available [1], [2], [3]. For conventional linacs, carbon fibre is the material of choice for the treatment couches, as it absorbs little dose and is structurally rigid [4], [5]. However, an electric current may be induced in carbon fibre due to its conductive properties, and it is therefore not suitable for use in an MRI scanner or an MR-linac. Instead, composite material couches have been developed for these machines. Thereby image artefacts due to the tabletop material are avoided, but the irradiation attenuation for these materials is generally higher than for carbon fibre. Also, they do not produce any signal on MRI, so the material information required for dose calculation cannot be derived from the images. As the effect of the patient positioning system (PPS) on the attenuation is significant, a model of the complete PPS structures must be included in the treatment planning system (TPS).

Due to the complexity of the PPS structures, there is a high variation in attenuation as a function of gantry angle, which might potentially result in a systematic error in the dose calculation for the beams passing through the PPS. For the same reason, the dose from a beam could be sensitive to lateral displacement. Furthermore, the presence of the very dense couch will affect the dose to the skin in contact with the couch, which is important to take into account in the dose planning[6], [7], [8].

Hence, the model of the MR-linac PPS structures in the TPS must very accurately represent the physical one. In effect, this also means that there should be very strict tolerance in the PPS structure production to be able to use a generic PPS model in the TPS across different machines. The present work presents a comparison of the measured and calculated radiation attenuation of the PPS structures of two high-field MR-linacs (Elekta Unity®) including detailed handling of measurement uncertainties

## 2 Materials and methods

### 2.1 The MR-linac patient positioning system

**Figure 1:**
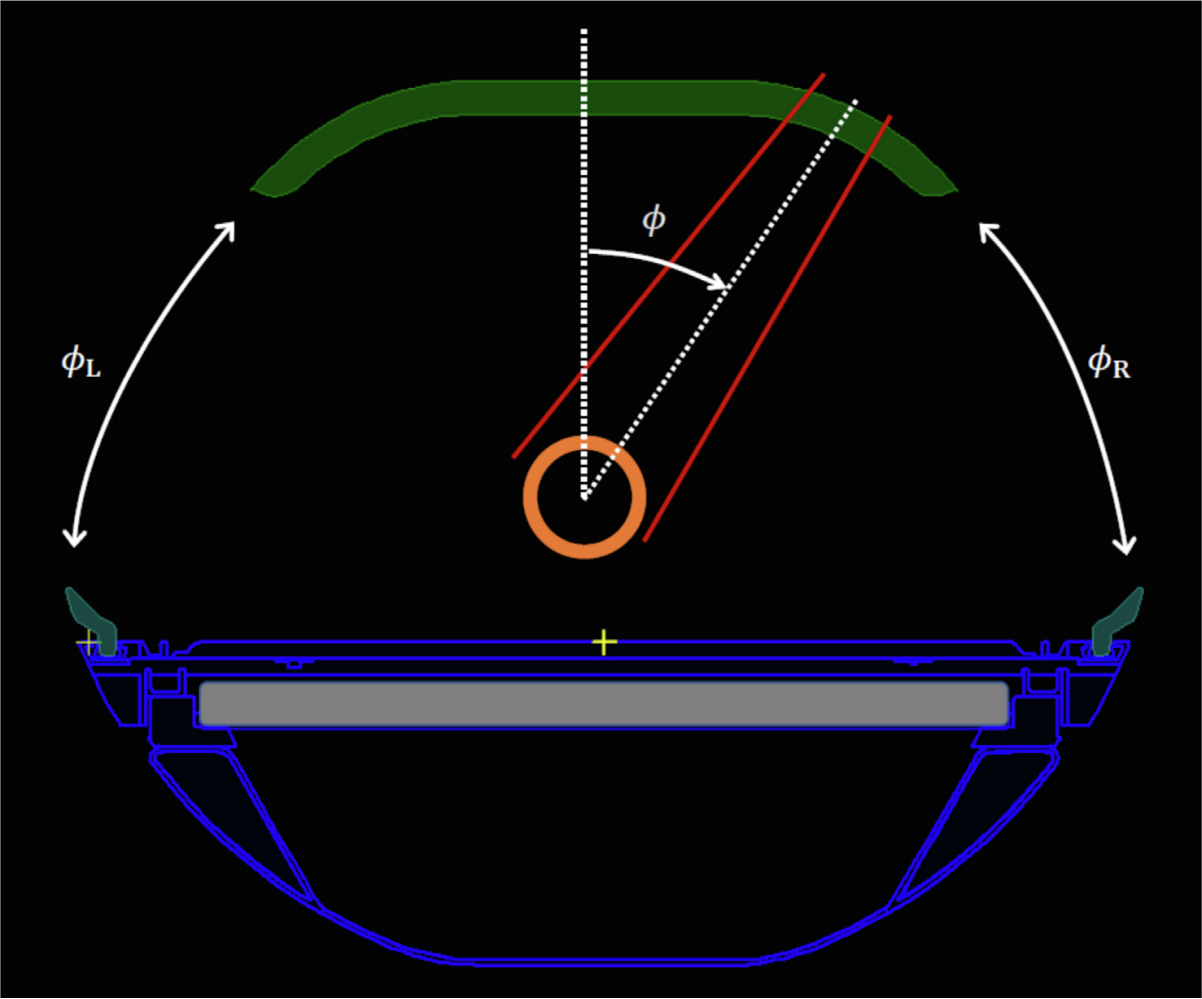
A screenshot from the TPS showing a cross-section of the Elekta Unity PPS. The major components are the complex couch structures (outer contours in blue) with varying internal densities, the anterior (green) and posterior coils (grey), and the phantom (orange). The gantry angle intervals where the beam does not pass any parts of the PPS are labelled $\phi_{L}$ and $\phi_{R}$. The red lines indicate the radiation field, and ϕ is the gantry angle.

The PPS consists of several structures with different materials and densities, see Fig. 1. The design constraints of the PPS were that it must be MR compatible and at the same time provide a stable couch top with a minimum deflection to ensure accurate patient positioning as well as uphold a specified distance to the posterior coil. Therefore, the structure with the highest mass density (and thus highest attenuation) is the couch top. On top of the couch is a thin comfort mattress for an improved patient experience. The posterior coil is mounted underneath the tabletop, and the anterior coil slides over the patient using sidebar rails.

The beam is also attenuated by the cryostat (CS) of the MR (not shown). The attenuations in the CS are individual to each MR-linac and thus individually modelled in the TPS in terms of a measured gantry angle-dependent attenuation lookup table. The CS consists of several materials including welded steel and aluminium cylinders and liquid Helium, but the exact materials are not disclosed by the vendor. A perfectly cylindrical CS filled with a sufficient amount of liquid Helium would exhibit a rotationally uniform transmission characteristic when rotating the gantry. However, the transmission varies with gantry angle, which is likely due to the welding seams in the CS.

### 2.2 Determination of the attenuation of the CS and the PPS

In this work, the attenuation was determined by the ratio of the dose to the detector (calculated or corrected measurement signal) with and without the attenuating element in the beam path. The measured signal was corrected for phantom temperature and air pressure. Other correction coefficients for transferring to the signal to dose cancel out since the ratio of the corrected reading is identical to the dose ratio. The ratio of the corrected reading is denoted by dose ratio, while the TPS calculations are based on calculated doses. The dose $D_{IC}$ (or signal) to the ionisation chamber (IC) placed at isocentre when irradiated from gantry angle ϕ can be described as

(1) $$D_{IC}\left(\phi \right)=D_{IC}\left(\phi =90^{{}^{\circ}}\right)T_{CS}\left(\phi \right)T_{PPS}\left(\phi \right),$$

where $T_{CS}$ is the transmission variation of the cryostat and $T_{PPS}$ is the transmission through the PPS. The normalisation of $T_{CS}$ to unity at angle $\phi =90^{{}^{\circ}}$ is chosen because the beam does not traverse any parts of the PPS (i.e. $T_{PPS}\left(\phi =90^{{}^{\circ}}\right)=1$).

The $T_{CS}\left(\phi \right)$ was determined through dose ratios from measurements with the PPS removed during the beam data characterisation and in the current work

(2) $$T_{CS}\left(\phi \right)={\left(\frac{D_{IC}\left(\phi \right)}{D_{IC}\left(\phi =90^{{}^{\circ}}\right)}\right|}_{No\;PPS}.$$

The dose to the IC with a uniform CS with no variation in the angular transmission is then

(3) $$D_{IC}^{Uniform}\left(\phi \right)=\frac{D_{IC}\left(\phi \right)}{T_{CS}\left(\phi \right)}.$$

The transmission through the PPS is defined as ratios of $D_{IC}^{Uniform}$ with and without the PPS in the beam path

(4) $$T_{PPS}\left(\phi \right)=\frac{{\left(D_{IC}^{Uniform}\left(\phi \right)\right|}_{PPS}}{{\left(D_{IC}^{Uniform}\left(\phi \right)\right|}_{No\;PPS}}.$$

As the isocentre of the MR-linac is located at 14.0 cm above the couch top, the gantry angle intervals where the beam does not encounter the PPS are $\phi_{R}≅\left[50^{{}^{\circ}},105^{{}^{\circ}}\right]$ and $\phi_{L}=360^{{}^{\circ}}-\phi_{L}≅\left[255^{{}^{\circ}},310^{{}^{\circ}}\right]$, see Fig. 1, where subscripts (R,L) indicate beam incidence from the right and the left, respectively, as seen standing at the foot end of the bore. At these angles, in theory, the PPS transmission is ${\left(T_{PPS}\left(\phi \right)\right|}_{\phi \in \phi_{R,L}}=\;1$ with $\phi_{R,L}=\left[\phi_{L},\phi_{R}\right]$. To avoid bias in $T_{PPS}$ due to noise for a specific angle, a mean value was taken over the $\phi_{R,L}$. Thus $T_{PPS}$ was calculated as

(5) $$T_{PPS}\left(\phi \right)=\frac{{\left(D_{IC}^{Uniform}\left(\phi \right)\right|}_{PPS}}{{\langle D_{IC}^{Uniform}\left(\phi \right)\rangle }_{\phi \in \phi_{R,L}}},$$

where the brackets 〈〉 indicate taking the mean. The attenuation of the PPS is then given as

(6) $$A_{PPS}\left(\phi \right)={1-T}_{PPS}\left(\phi \right).$$

For all measurements and calculations in the current work, a 7 megavoltage (MV) flattening filter-free (FFF) beam with a field size of 5×5 cm^2^ and 100 MU was used to irradiate the phantom. The gantry angles were in steps of 1° steps from 90° to 270° and 5° steps from 275° to 90°, altogether 214 gantry angles. The gantry angles of 10° and 15° were not allowed due to the superconducting pipe connecting the two magnet segments. Irradiation of the superconducting pipe is avoided on the Unity and in TPS. The forbidden gantry angle range depends on the lateral field size, e.g. symmetric lateral field size of 5.0 cm and 10.0 cm, the forbidden angle ranges are from 8.8° to 17.8°, and from 8.0° to 18.7°, respectively.

### 2.3 The PPS attenuation experimental setup

**Figure 2:**
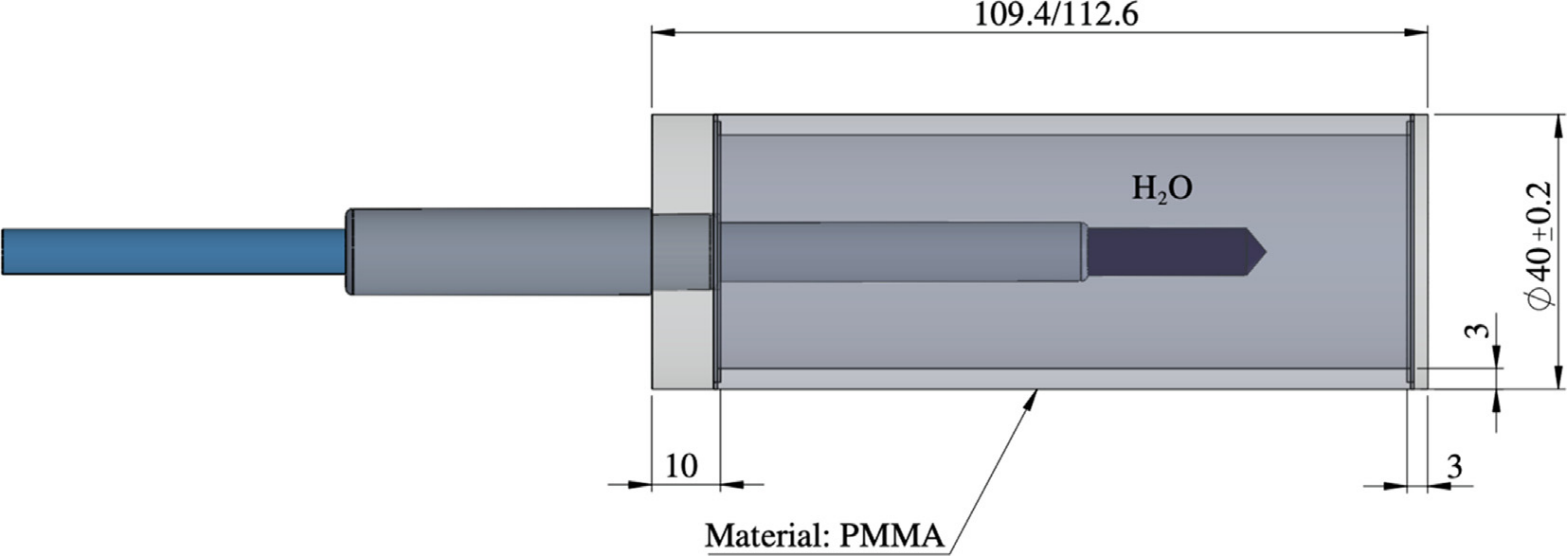
The water-filled PMMA phantoms used in these experiments with sizes all given in mm. The only difference between P1 and P2 is the length (109.4 and 112.6 mm, respectively). The IC is inserted and fastened in the hole on one side of the cylinder.

**Figure 3:**
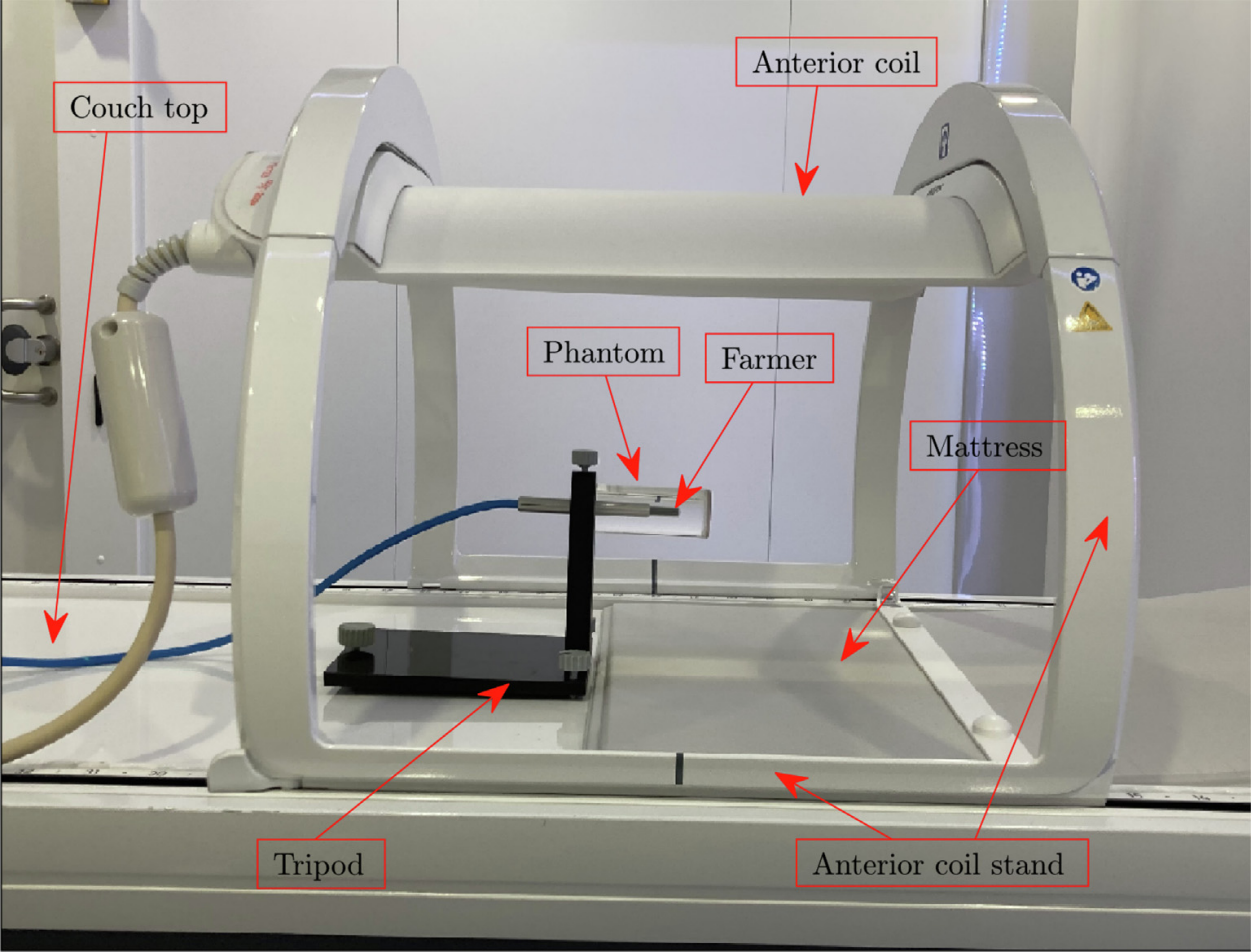
The phantom setup outside the bore is shown. The Farmer chamber is mounted into the water-filled cylindrical phantom, which is held by an adjustable tripod. The anterior coil is mounted in the highest position above the phantom. The end of the mattress is seen to the right of the tripod and below the sensitive volume of the Farmer chamber.

Two Elekta Unity® (Elekta, Stockholm, Sweden) high-field 1.5 T MR-linacs installed in 2018-2019 at Odense University Hospital, Odense, Denmark (OUH) and Uppsala Akademiska Sjukhus, Uppsala, Sweden (UAS) were investigated. A cylindrical water phantom was manufactured from polymethylmethacrylate (PMMA), with an outer diameter Ø4 cm, an outer length 11 cm, and a wall thickness 3 mm, see Fig. 2. The phantom cavity was filled with distilled water in order to minimise the effects of air cavities[9]. Farmer ion chambers of types PTW (PTW-Freiburg, Freiburg, Germany) 30006 (OUH) and PTW 30013 (UAS) were positioned in the phantom. The ICs were connected to electrometers, PTW UNIDOS webline and Scanditronix-Wellhöfer, Dose1 (IBA Dosimetry GmbH, Schwartzenbruck, Germany) at OUH and UAS, respectively. Electrometer readouts were corrected for air pressure and temperature but were not compensated for a possible drift in the MR-linacs. However, by repeating the same measurement during the course of a session, the magnitude of drift could be monitored. The phantom as well as parts of the PPS are depicted outside the bore in Fig. 3.

Some measurements were carried out manually per gantry angle, i.e. manually starting the electrometer and registration of the electrometer readings into a spreadsheet. To make this more automatic and less prone to manual errors, in-house software was developed to read out the electrometer reading to a spreadsheet. This was based on the autostart function in the PTW Unidos webline electrometer, which resets and starts a measurement when the signal is above a set sensitivity (low) threshold.

The data were collected with two different phantoms P1 and P2 (differing in length); see dimensions in Fig. 2. In the measurements with P1, a small amount of isopropyl alcohol was added to avoid air bubbles in the distilled water inside the phantom. Although it worked well, it also resulted in cracks in the PMMA material after a few series of measurements carried out over a period of approximately two months. Therefore, the phantom P2 replaced P1 and the use of isopropyl alcohol ended.

The alignment of the Farmer chamber relative to the cylindrical axis of the phantom depends on the production accuracy, such as centring the hole to the axis of the phantom. Also, the degree of tightening of the Farmer chamber into the phantom might impact the alignment precision of the Farmer chamber relative to the phantom.

The anterior coil was elevated to its maximum position with the coil edges 31.5 cm above the couch top. The IC was positioned with the chamber reference point (CRP) at the radiation isocentre with only the help of the onboard megavoltage imager (MVI, i.e. the electronic portable imaging device). The CRP of the IC was set on the inner electrode at 13 mm from the tip of the outer electrode. While the inner electrode of the chamber was well visualised on the MVI, the outer tip was obscured by image noise. The Farmer chamber was aligned relative to the MV isocentre by a projection of two orthogonal beams onto the MVI panel. A red cross marker available in the MVI software (MVIC) was used to mark the position of the MV isocentre in the MVI images. Thus, a setup uncertainty of ±1 mm in the longitudinal direction and cross-plane (plane perpendicular to $B_{0}$) < ±0.5 mm was estimated for subjective MVI analysis by the operator.

The IC orientation was oriented anti-parallel to the magnetic field $B_{0}$ of the MR-linac[10]. The IC marker was oriented either up (manual measurements) or left and right (auto acquisition measurements using in-house developed software).

The effect of setup errors and different sources of uncertainties were investigated. These included chamber and phantom isocentre offsets, rotational asymmetry of the IC response, rotational misalignment of the IC and phantom unit, the effect of the phantom length, and variations in the MR-linac output. The headlines of these investigations are given in a summary format under the results section 3.4.

### 2.4 Compensating for the sinusoidal variation in the measurement

#### 2.4.1 Development of compensation of sinusoidal output based on OUH CS data

The vendor performed the initial CS characterisation during the beam data commissioning of the TPS. The experimental setup at that time was based on a solid cylindrical PTW build-up cap for the Farmer chamber insert on the cylindrical axis with an outer diameter of 56.5 mm. The PPS was removed, and a special Elekta holding device positioned the Farmer and the build-up cap with the CRP at the isocentre of the MR-linac. Several beams were delivered at different gantry angles with a field size of 10×10 cm^2^.

However, the vendor characterisation of the CS was carried out differently at the two centres. Multiple measurements with rotation of the solid build-up cap and the Farmer chamber were performed at UAS, whereas a single measurement was performed at OUH. Air cavity effects that are sinusoidal in nature and periodic over a $360^{{}^{\circ}}$ chamber and build-up cap rotation may occur in solid phantoms[9], [11]. Therefore, the characterisation of the OUH CS was checked by repeating the measurements using the cylindrical water phantom described in this work

A compensation strategy was developed to mitigate the variations in the measurements due to sinusoidal effects, e.g. air cavities and minor setup errors, propagated to the attenuation calculations of Eqs. (2)-(6). Note that Eq. (3) is a ratio and requires that the sinusoidal variation is the same, but not necessarily zero, and thus it is sufficient to minimise the difference in the sinusoidal variation between two measured data sets. Moreover, since $T_{CS}^{M}$ and $T_{CS}^{ref}$ are normalised to unity at ϕ=90^°^, the difference will vanish there. So in this work, we propose that the difference in sinusoidal variation between two measurements $T_{CS}^{M}$ and $T_{CS}^{ref}$ of the CS, transmission can be modelled by a cosine function of the form

(7) $$a\left[\cos\left(\phi -b\right)-\cos\left(90^{{}^{\circ}}-b\right)\right]\;,$$

where a and b are constants to fit measurements $T_{CS}^{M}$ to the sinusoidal behaviour of $T_{CS}^{ref}$. The constants a and b are found using the least-squares method[12] (LSM) by

(8) $$\left\{a_{CS},b_{CS}\right\}=\min_{a,b}\sum_{\phi_{i}\in \phi_{all}}{\left\{T_{CS}^{M}\left(\phi_{i}\right)-T_{CS}^{ref}\left(\phi_{i}\right)-a\left[\cos\left(\phi_{i}-b\right)-\cos\left(90^{{}^{\circ}}-b\right)\right]\right\}}^{2},$$

where $\phi_{i}$ is a member of all discrete gantry angles $\phi_{all}$. Thus, the measured CS transmission after compensation for sinusoidal variation artefact with $T_{CS}^{ref}$ as a reference, according to Eq. (8), is written as

(9) $$T_{CS}^{fit}\left(\phi \right)=T_{CS}^{ref}\left(\phi \right)+a_{CS}\left[\cos\left(\phi -b_{CS}\right)-\cos\left(90^{{}^{\circ}}-b_{CS}\right)\right].$$

These sinusoidal effects is a phase shift of the transmission data, and that's what is compensated, e.g., the phase shift $b_{CS}=0^{{}^{\circ}}$ then $a_{CS}=0$. Thus, no correction if there is no phase shift. Furthermore, the denominator in Eq. (5) reduces the random error (noise) in the data. The effect of the compensation will be illustrated on the CS measurement, and then a similar procedure will be applied to the PPS measurement for angles where no PPS is in the beam path, as will be explained in the next section.

#### 2.4.2 Compensation of previous CS characterisation to the PPS measurements

Measurements of the CS $T_{CS}^{M}$ for $\phi_{L,R}$ with the PPS in the bore are used to compensate the TPS version $T_{CS}^{TPS}$ towards the new measurement $T_{PPS}^{M}$. The LSM was again used to match $T_{CS}^{TPS}$ to the measurements in the actual setup with the same compensating function as used in the previous section. The coefficients $a_{PPS},b_{PPS}$ of the compensating function were found as

(10) $$\left\{a_{PPS},b_{PPS}\right\}=\min_{a,\;b}\sum_{\phi_{i}\in \phi_{L,R}}{\left\{T_{PPS}^{M}\left(\phi_{i}\right)-T_{CS}^{TPS}\left(\phi_{i}\right)-a\left[\cos\left(\phi_{i}-b\right)-\cos\left(90^{{}^{\circ}}-b\right)\right]\right\}}^{2}.$$

The transmission of the PPS is calculated by using Eqs. (5), (6) where the $D_{IC}^{Uniform}\left(\phi \right)$ is calculated using Eq. (3) with the $T_{CS}\left(\phi \right)$ being replaced by the adapted CS transmission $T_{CS}^{ADA}\left(\phi \right)$ obtained from Eq. (10) as

(11) $$T_{CS}^{ADA}\left(\phi \right)=T_{CS}^{TPS}\left(\phi \right)+a_{PPS}\left[\cos\left(\phi -b_{PPS}\right)-cos\left(90^{{}^{\circ}}-b_{PPS}\right)\right].$$

### 2.5 The calculation of the PPS attenuation in the TPS

One set of calculations of the attenuation was based on the PPS model extracted from the TPS in clinical use (Monaco® v5.4) for the Elekta Unity MR-linac (hereafter called v5.4). As v5.4 has been shown to slightly overestimate[13], [14] the attenuation from gantry angles around 180°, an improved voxelisation was also tested using a development version of Monaco v6.x (exact version still not known) for the upcoming release for gating and hereafter called Monaco Dev or simply Dev. The procedure to assign densities to the dose calculation voxels is described in the TPS manual. In the case of regions with assigned densities (such as the PPS), the TPS starts by determining which image voxels belong to the region. Then the dose voxels are assigned a density by subsampling in the image voxel grid. In v5.4, there is a pre-processing step of moving the contours such that they are aligned with image pixel edges, before assigning densities to the image voxels, and this was biased towards enlarging small structures. This pre-processing was removed in Dev together with introducing an improved algorithm for interpolating in the image voxels when assigning density to the dose voxels^1^.

The resulting voxelised models of the phantom and PPS from both TPS versions were used for dose calculation performed in a research version of the TPS using the respective clinical beam data from UAS and OUH. The detector (PTW Farmer 30006/30013) was modelled by 5×12 (5×8) voxels for a 2 mm (3 mm) dose calculation grid to approximately match the sensitive volume with a radius of 3 mm and a length of 23 mm. The five voxels consisted of the centre voxel and its four neighbours (up, down, left and right). The calculated dose to the detector was the average dose over the voxels belonging to the detector. The attenuation was subsequently calculated using Eqs. (5), (6).

The geometry of the phantom was determined from computerised tomography (CT) scans in a setup equivalent to the measurement setup on a Philips 16 slice Big Bore Brilliance scanner (Philips Medical Systems, Best, The Netherlands). The phantom was modelled as homogeneous liquid water with the detector at the isocentre. The same field size and monitor units (MU) were used for the measurements (5×5 cm^2^, 100 MU).

The finer structures of the PPS are subject to discretisation effects when converting the PPS model to voxels with material and density in the dose calculations. Therefore, the calculations were repeated for different calculation grid voxel sizes (2×2×2 mm^3^ and 3×3×3 mm^3^) and underlying CT image resolution (0.5×0.5×1 mm^3^ and 1×1×1 mm^3^). The noise was set to 1% per voxel in the Monte Carlo dose engine.

## 3 Results

### 3.1 Compensation of the sinusoidal artefact of the CS attenuation measurements

**Figure 4:**
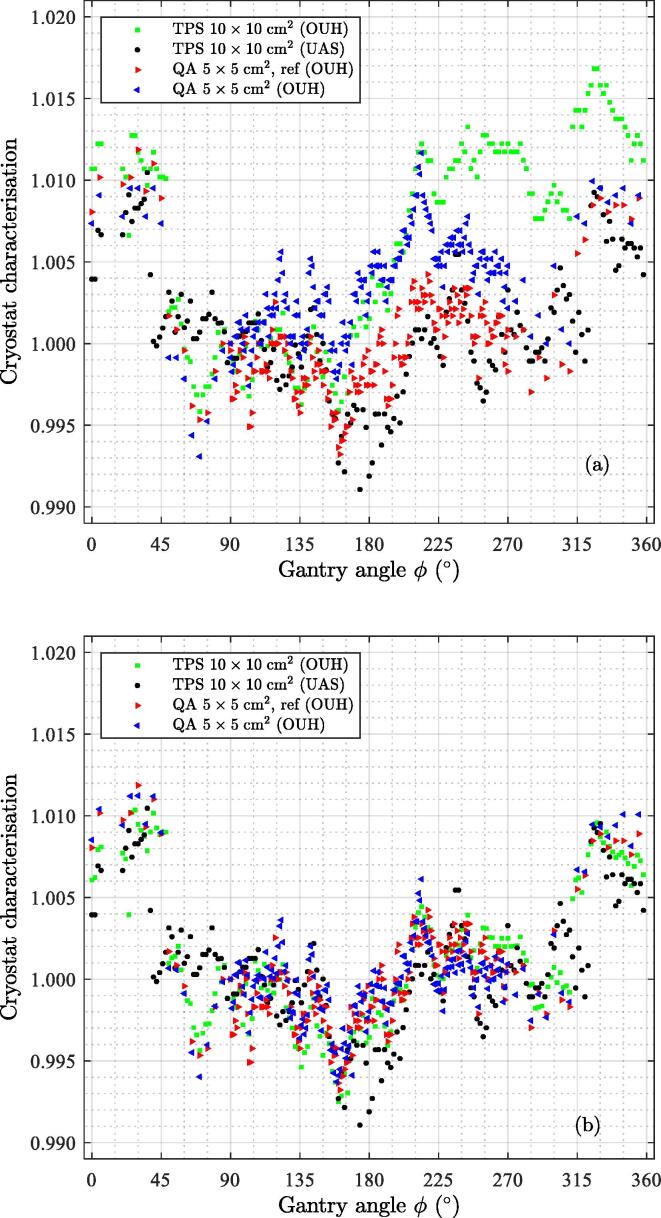
Normalised CS attenuation for the MR-linacs at OUH and UAS. The normalisation refers to the value at a gantry angle of 90°. The normalised CS attenuation is a part of the TPS and was measured during this work (OUH). (a) The raw measured normalised data. The mean standard deviation (SD) across the gantry angle of the OUH data is 0.003; (b) The OUH normalised CS data compensated towards the reference data points labelled ref. The mean SD of the OUH data reduces to 0.001.

The procedure to compensate for the sinusoidal variation artefact was tested on the CS data, measured during the beam data collection and in the current work at OUH. The CS characterisation $T_{CS}\left(\phi \right)$ of the two MR-linacs are shown in Fig. 4. The variation in the transmission is less than 1% except for the measurement performed at OUH during the beam data collection, where no compensation for rotational air cavity effects was performed. The compensation for the sinusoidal variation artefact, using the QA ref (OUH) data as reference (see Eq. (9)) reduces the spread of measured TPS (OUH) and QA (OUH), see figure caption of Fig. 4. No difference in the CS characterisation for the field sizes 5×5 cm^2^ and 10×10 cm^2^ could be detected.

### 3.2 The measured PPS attenuation at OUH and UAS

**Figure 5:**
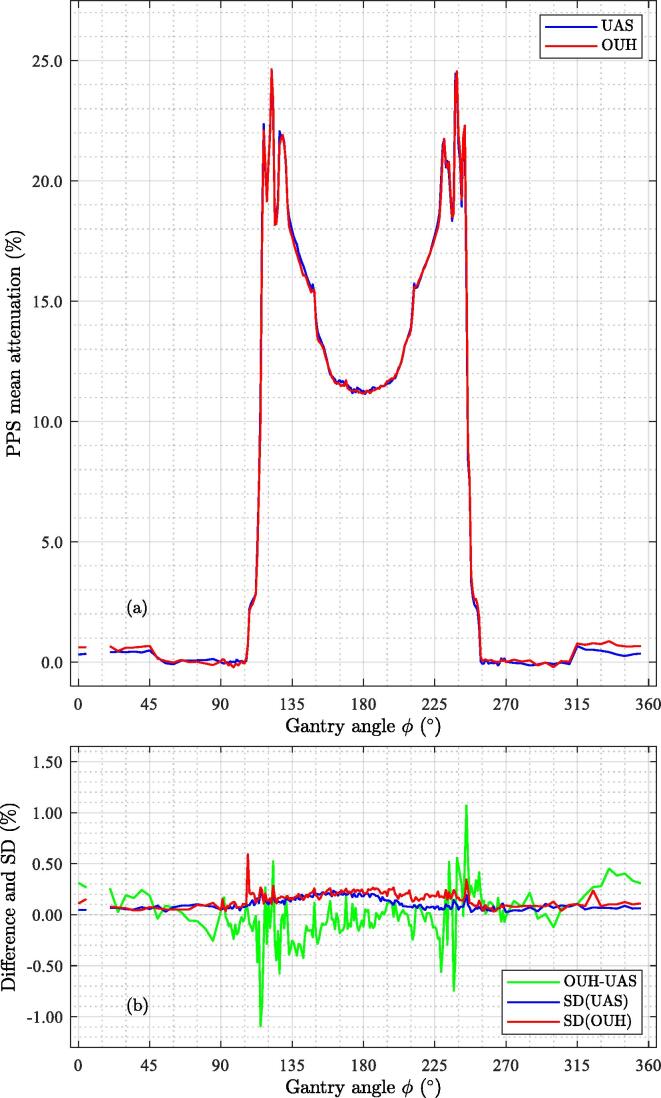
Measured attenuation curves for the two PPSs. (a) Attenuations; (b) Differences as well as the measurement uncertainty given as 1 standard deviation (SD).

**Figure A.1:**
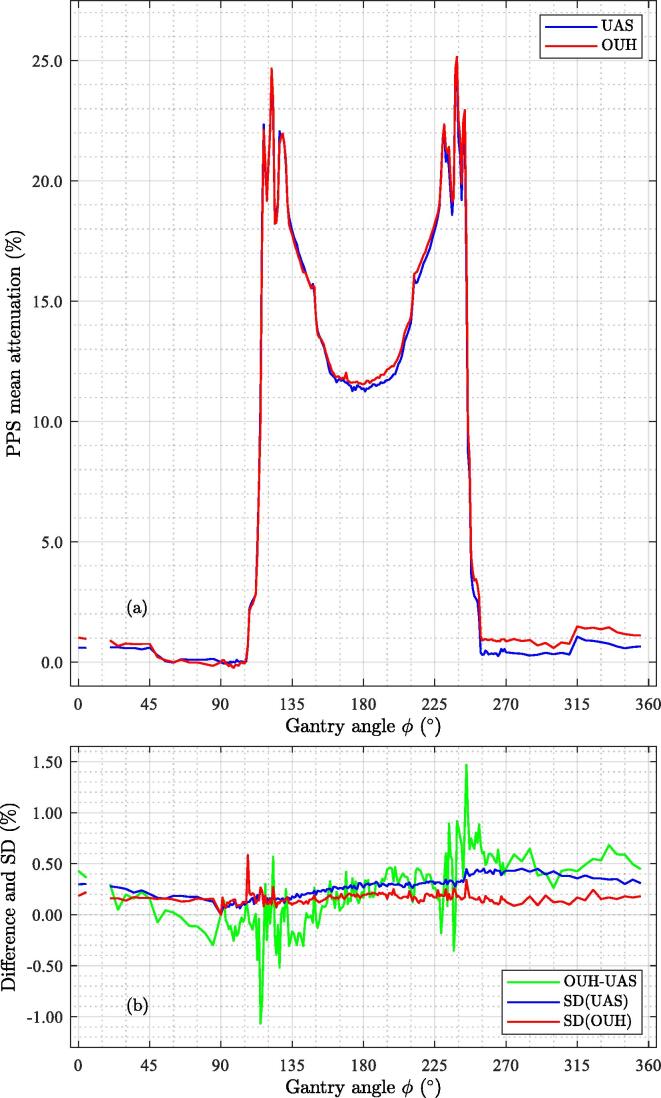
The PPS attenuation based on the raw data without compensating for the sinusoidal variation. (a) PPS mean attenuation. There is an apparent effect on the OUH mean attenuation seen for gantry angles starting from 180° and increasingly pronounced for higher gantry angles; (b) Difference and SD. The SD of the UAS data is slightly larger than the OUH data in a wide gantry angle range.

No significant difference between the data obtained manually and automatically was found, and there will be no distinction between manual and auto acquired data henceforth. The measured attenuation, normalised at 90° after applying the compensation for the sinusoidal variation artefact is shown in Fig. 5. As can be seen, the attenuation of the PPS at the two sites is very similar. The effect of the compensation procedure is visible in the attenuation result before and after the compensation, as can be seen in Fig. A.1 in Appendix A.

Very steep gradients in the attenuation (0% to 25% in a 15° range) occur when the beam traverses the complex structures on the sides, high-up, close to the couch top (cf. Fig. 1) at gantry angles 112°-127° and 232°-247°. The peak attenuation for both PPSs occurred at gantry 122° (24.6% at both sites) and 238° (24.6% and 24.5% for OUH and UAS, respectively). The maximum attenuation in the anterior coil was 0.69% (UAS) and 0.88% (OUH). The attenuation at 180° was 11.3% for both UAS and OUH. The differences in attenuation were mainly within ± 0.5% with the difference of –1.1% at gantry angle 115° and 1.1% at gantry angle 245°. The measurement uncertainty is approximately 0.25% (1 standard deviation (SD)) except for a small gantry angle interval related to the steep attenuation gradient.

### 3.3 Comparing the calculated and the measured PPS attenuation

**Figure 6:**
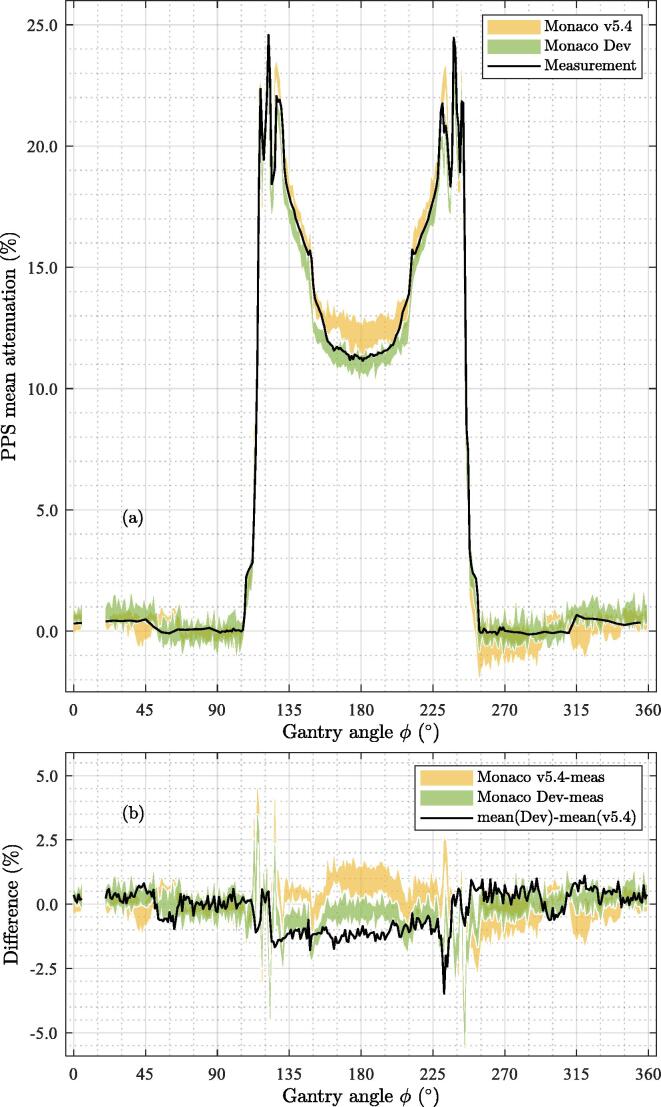
Comparison of calculated and measured attenuations. (a) Calculated attenuations (Monaco v5.4 and Monaco Dev) and the measured (meas as the mean of OUH and UAS shown in Fig. 5) as a function of gantry angle. The calculated attenuation per voxelisation was averaged over OUH and UAS. Presented here is the range over the four different voxelisations; (b) The difference between the computed attenuation and the average measurement, and the mean of the difference between v5.4 and Dev.

A noticeable improvement in the dose calculation accuracy for angles 160°-200° can be seen from version v5.4 to Dev. The differences between measured and calculated attenuation are generally within 1.0% for Dev and 1.0 - 2.0% for v5.4, see Fig. 6. The SD of the calculations was in the range [0.21%-0.35%] over the different voxelisations for v5.4 and Dev as calculated using the open-angle ranges $\phi_{L,R}$. The most significant deviations are for 10° intervals around angles 120° and 240°, where the error can be up to 4-5%.

### 3.4 Sensitivity analysis of the results with respect to measurement uncertainties

Several experiments were performed to investigate the robustness of the experimental results. One of the investigations was carried out on an Elekta Versa HD linac (explicitly mentioned in the text), while all others were on the MR-linac. The results of these investigations and sources of uncertainties are briefly reported here. The first three are candidates for contributing to the sinusoidal variation in the measurements and are thus handled by the presented compensation method.

**Figure B.1:**
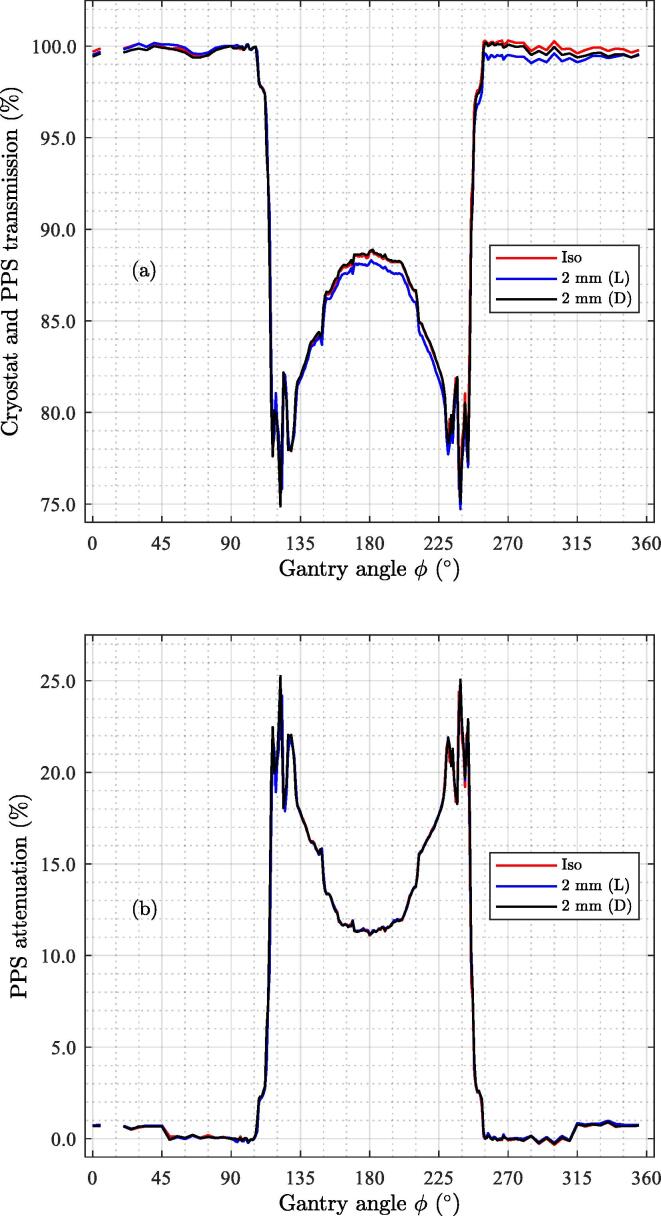
Three measurement series with the detector in three different positions. (a) The measured attenuation with a 2 mm displacement of the IC laterally (L) or vertically downwards (D), compared to when the detector was in the isocentre (Iso); (b) The attenuation from the same measurements after sinusoidal compensation.

• **Lateral and vertical misalignment of chamber and phantom** was investigated through displacements of the phantom (and detector) relative to the isocentre. A 2 mm displacement towards the patient's left (in Head First Supine position) in ∼1% difference in the measured dose. The sinusoidal compensation was able to recover the attenuation without displacement, see Fig. B.1 in Appendix B. This is an indication that the compensation strategy is meaningful. The effect of a 2 mm displacement down towards the couch top was negligible.
• **Rotational asymmetry of the IC response** was investigated by rotating the chamber around its symmetry axis without a build-up cap 360° in the 6 MV FFF beam of a conventional linac (Elekta Versa HD®). The difference was slight and within 0.1%.
• **Rotational misalignment of the IC and phantom** was investigated by rotating the whole unit around the outermost metallic cylindrical part of the IC in steps of 22.5° and repeating the measurements. The difference was within 0.2%.
• **Longitudinal position misalignment** was evaluated by moving the phantom ±1 cm (in/out of the bore) relative to the isocentre in steps of 1 mm. Beams of 5×5 cm^2^ and 100 MU were delivered in turn at gantry 90° and 180° degrees five times. A misalignment of 5 and 10 mm yields an error in attenuation of 0.1% and 0.2%, respectively and thus negligible.
• **Effect of phantom lengths** (P1 and P2 not the same length) was investigated by adding a polyoxymethylene disc of a thickness of 1.0 cm and an outer diameter of 4.0 cm to the tip of P1. No difference in the ratio of measured data at gantry angles 90° and 180° could be detected.
• **Variation in MR-linac output.** The variation in machine output at gantry angles 90° and 270° was 0.15% (1 SD). The drift in MR-linac output, as measured by the difference in the collected charge in the first and last measurement, dropped around 0.33-0.40%.

## 4 Discussion

In the current work, we have made measurements of the CS transmission (at OUH) and the PPS attenuation characteristics to compare the MR-linacs at our two installations as well as validate the PPS modelling in both a clinical and an upcoming version of the TPS.

The measurements show that the attenuation of the PPS varies strongly as a function of gantry angle with peaks up to 25% at two gantry angle regions 112°-127° and 232°-247°. Our experiments show that a misalignment corresponding to a change of 1° gantry angle could result in as much as ∼1.6% difference in attenuation in these particular angle intervals, see Fig. 5. So consideration should be given to this when these gantry angle ranges are utilised in a treatment plan. The difference in attenuation between the two installations was very small, generally within 0.5% with maximum differences of 1.1%. This supports that a generic PPS model can indeed be used in the TPS. The overall measurement uncertainty in the attenuation was generally 0.2% with an exception for a few angles with complex attenuation patterns.

The calculation of the attenuation of the PPS in the TPS agrees with the measurement in general within 1% for most gantry angles. Hence, the dose calculation accuracy of the couch and coil model in the TPS is at the same level. However, the gantry angle ranges with the high gradient in attenuation with respect to gantry angle show differences between calculations and measurements of up to 4-5%. This underlines that consideration should be taken when using these angles in clinical practice. The systematic over estimated attenuation by the TPS (v5.4) of the attenuation of ∼1.5% from gantry angles around 180° was improved in the development version of the TPS (Dev) where an improved voxelisation algorithm was implemented. The different voxelisations, arising from a different dose grid resolution and a CT image pixel size, resulted in a range of 1% in the calculated attenuation.

The sinusoidal variation seen in the CS model from the beam data characterisation, due to the presence of air cavities[9], [11] and/or setup uncertainties between the Farmer chamber and the solid PMMA phantom, was possible to compensate using the presented method. Data in the literature are (0.990-1.025)[15] and (0.985-1.003)[14] agree well with our findings, see Fig. 4, which had the range (0.991-1.011, TPS, UAS) and (0.996-1.017, TPS, OUH) when normalised to unity at gantry angle 90°. The range after applying the introduced compensation was (0.993-1.010, OUH).

A series of tests were made to assess the measurement uncertainties such as machine output stability, rotational asymmetry of the detector response, rotational and longitudinal misalignment of the phantom and detector, and the effect of the small difference in phantom length. It was also shown that the PPS attenuation was insensitive to cross-plane misalignment when the rotational asymmetry correction procedure was applied. The repeatability of the measurements was shown to be approximately 0.25% (1 SD) for most gantry angles and within 0.5% for all. Thus, when all uncertainties are considered, we claim that the measurement uncertainty of the results is within 0.5%.

## 5 Conclusions

This work shows that the two MR-linac PPSs have only minor differences in attenuation. Mean differences exceeding ±1% were observed at around gantry angles 115° and 245° where the attenuation pattern of the PPS is very complex and a steep gradient in the attenuation was seen. The difference between measured and calculated attenuation by the TPS was generally found to be within 1-2% in the clinical TPS and improved to ±1% for a development version of the same TPS. For both models, larger differences were found at the angles with complex attenuations and in the vicinity of 180°.

Avoiding irradiation through the complex parts of the PPS with a steep attenuation gradient would be preferable in clinical situations if possible. The gantry angles at which irradiations of the complex parts occur depend on the lateral field size and the position of the target volume in the patient planned for radiotherapy

## Declaration of Competing Interest

The authors declare that they have no known competing financial interests or personal relationships that could have appeared to influence the work reported in this paper.

## Appendix A PPS transmission using TPS CS data

If the compensation of the sinusoidal effect was omitted in the data shown in Fig. 5, the PPS transmission would appear different. The PPS transmission with no compensation is found as measured raw data divided by the TPS CS characterisation. The result is displayed in Fig. A.1.

## Appendix B Evaluation of the proposed compensation procedure

A test was carried out to validate the compensation procedure. A small chamber offset away from the isocentre was applied and compared to no offset. The results are shown in Fig. B.1.

## Footnotes

1 Note that v6.x Dev and the latest clinical release v5.51 (not used in the current work) share that there is no pre-processing of the contours - which means that the calculations in Dev resemble those in v5.51. However, v5.51 and Dev do not share exactly the same algorithm when interpolating in the image voxels to assign electron densities to the dose calculation voxels[16]. Thus the result of the current work could differ slightly to results from v5.51.
